# New York State Medical Association

**Published:** 1886-01

**Authors:** 


### NEW YORK STATE MEDICAL ASSOCIATION.

At the second annual meeting of the NEW YORK STATE MEDICAL ASSOCIATION, in New York, on November 17th, 18th and 19th, the President, John P. Gray, in his address discussed the question of the *Power and the Right of the State to License Medical Practitioners*, and took the ground that as the state must necessarily recognize all "schools and systems" of medicine, legislative interference is not called for, either in the interest of medical science, or of the medical colleges.

Dr. Alfred L. Carroll, of Richmond County, delivered an address on *State Medicine.* State medicine includes medical jurisprudence, medical education and public hygiene. The latter is held by the public as the most important, yet the medical student receives little or no instruction in this branch, and the profession at large has a deplorable lack of knowledge upon this subject.

Dr. Henry Didama, of Onondaga County, said of *Tubercular Consumption—Is it Ever Inherited?* The speaker assumed the infectiousness of the disease and its dependence upon the presence of the bacillus tuberculosis. He reviewed the literature upon the subject, and described an epidemic of the disease among cattle in which young calves, and even fœtuses, were found affected. Similar instances of hereditary transmission have not been found in the human subject. His conclusions are that (1) in man tubercular consumption is not hereditary; (2) that there is no peculiar hereditary tendency; but (3) children of tuberculous parents, as well as those of other parents debilitated by disease, may be weak and may easily acquire tuberculosis. They are more likely to become affected if they live among tuberculous than if they live among non-tuberculous individuals. In the discussion Dr. Rochester, of Erie County, related a case in which he found tubercles and a cavity in the lungs of a child three weeks old. Its mother was healthy, but its father had died of tuberculosis before the child was born. The speaker believed tuberculosis may be hereditary.

Dr. Simeon Clark, of Niagara County, read a paper on *Psöitis and Peripsöitis.* After reviewing the pathology and differential diagnosis he related three cases.

Dr. Hyde, of Cortland County, said that acute abscesse were at first not connected with the vertebræ; that caries of the spine is secondary.

Dr. Charles Brown, Chemung County, read a paper on *Shock, and the Effects of its Injuries upon the Nervous System*, describing the different forms and manifestations of shock and the conditions modifying it.

Dr. Frank Hamilton, of New York City, read a short paper on *Railroad-Shock*. He recognized two varieties. The first manifests early symptoms and is true shock. The second presents no early symptoms; is not true shock; there is primarily no injury to the spinal cord, but the ligaments of the spine are injured. This results in inflammation, which, by extension, leads to meningitis and myelitis. He ascribes the cause of the injury to sudden pitching forward of the body and its immediate recoil, like the snapping of a whip-lash.

Dr. Edmund S. F. Arnold, of New York City, read a paper on *The Essential Nature of Shock*. He considers it due to an injury to the centers controlling nutrition and to the partial arrest of nutrition.

Dr. Charles F. MacDonald, of Cayuga County, reported a case of gun-shot wound of the head, followed by insanity. The patient had been wildly maniacal for weeks following the injury. It was proposed to trephine. On reaching the bone it was found already open. With a hypodermic needle a cyst was discovered and evacuated. On recovery from ether the patient was perfectly rational.

There was a *Discussion on Pneumonia*, under the following heads:

*Question* 1. Is acute pneumonia a primary local inflammatory disease, or is it an essential fever?

Dr. Didama said that there have been three views as to the pathology of this disease, viz.: (1) that hyperinosis (excess of fibrin in the blood) is its chief characteristic; (2) that congestion of the vessels of the lungs from exposure of the surface of the body to cold is the principal exciting cause—pneumonia being considered an ordinary inflammation; (3) that it is an "essential fever," having a local lesion as secondary.

Dr. Ross favored the supposition that pneumonia was a general not a local disease, because true croupous pneumonia could not be set up by artificially irritating the lung tissue. Besides, the prostration accompanying the disease was out of proportion to the tissue invaded, and, moreover, there is a selection of a particular point, the right lower tube, for the local manifestation as in other general fevers.

*Question 2.* What facts can be cited in support of the doctrine that acute lobar pneumonia depends on the presence of specific micro-organism?

Dr. Janeway gave a review of the investigations hitherto made on the micrococci (pneumococci) of the sputa from a pneumonic lung.

By inoculation these micrococci will give rise to pneumonia in rabbits or guinea-pigs. The usual mode of infection is by the respiratory tract.

*Question 3.* What conditions or circumstances incident to acute lobar pneumonia render the disease fatal?

Dr. Robb stated that pneumonia is more fatal to negroes than to whites and more to women than to men.

Involvement of the upper lobes adds greatly to the danger. Alcoholic habits in the individual diminish or destroy his chance of survival. Some epidemics are milder than others.

Among the symptoms indicating great prostration and probability of a fatal issue are a temperature of 105° and upward, prune-juice expectoration, active delirium or, still worse, a low muttering fever and subsultus.

Dr. Biggs stated that only 6 per cent. died in 600 cases of uncomplicated pneumonia. Unfavorable prognosis was given in the following classes of cases: (1.) Patients of bad habits, particularly drinking men; also old persons. Patients who in the height of the fever have to be transported (as to a hospital) are very likely to die from the effects of this moving. (2.) Cases in which other affections co-exist, *e.g.*, Bright's disease, valvular disease of the heart, or surgical operations. (3.) Cases complicated by abscess, purulent infection, pleurisy with effusion, peri-and endo-carditis. (4.) Cases of pneumonia affecting more than a single lobe. (5.) Cases of great intensity, such as are frequent in some epidemics.

*Question 4.* Are there any remedies known capable of arresting or shortening the course of pneumonia or conducing to a favorable termination?

Dr. Rochester stated that formerly he had used the lancet and calomel with as good results as any since obtained in his practice. Ammonium carbonate, in five to ten grain doses, given in milk, was also recommended. Veratrum and other cardiac or arterial sedatives were not approved, nor was alcohol, except in asthenic cases.

*Question 5.* Is blood-letting ever indicated, and, if so what circumstances are there indicating and contra-indicating its employment?

Dr. Clark believed bleeding would relieve certain plethoric cases in which there was cardiac exhaustion, and dilatation of the right ventricle, and in which stimulants failed to secure improvement. Careful selection of the cases suitable for blood-letting was important.

*Question 6.* Is alcohol useful and what are the indications, doses, etc.?

Dr. Shrady thought alcohol was over-rated in medicine. It has little effect upon the pyrexia. Old people need stimulants when attacked with pneumonia.

Dr. Ferguson said alcohol was doubtless a true food, although much overestimated by medical men who had only vague ideas of its action. Temporarily it increases the action of the heart yet it acts as a sedative.

*Question 7.* To what extent is it safe and useful to employ antipyretic measures in acute lobar pneumonia inclusive of the cold-bath, sponging the body, or the wet sheet?

Dr. Stockton believed these measures, when found to be well borne by the patient, were valuable, but when seemingly ill-borne, were dangerous. Cold-sponging he considered to be the only safe measure. More than gr. xx of antipyrin should not be given in one dose, and this was not to be repeated until eight or ten hours had elapsed.

*Question 8.* Do relapses frequently occur after convalescence?

Dr. Orton had not met with relapses, and he thought that those so-called were really due to the extension of the disease to new tissue. Bronchitis, carditis, nephritis, etc., were liable to appear as complications, but not often meningitis. Second attacks of pneumonia or pulmonary phthisis were not uncommon sequelæ.

Dr. Van Dewarker presented a paper on *The Medico-Legal Bearing of Pelvic Injuries in Women.*

In cases of actions involving these injuries the defense was at a disadvantage since it could not procure evidence of the real trouble, the court being averse to ordering an examination of the women. Many fraudulent cases were trumped up by women. The law should compel an examination to be made in these cases.

Dr. A. Flint, Jr., delivered an address entitled *Some of the Relations of Physiology to the Practice of Medicine.*

A strong plea for accurate physiological knowledge, rather than empiricism, as the basis of medicine was made. The study of cardiac diseases was used to illustrate this claim. The investigation of micro-organisms was pointed out as an illustration of the superior value of scientific physiology in helping practical medical knowledge.

Dr. Taylor read a paper on *Recto-Labial or Vulvar Fistulæ—Their Causes and Treatment.*

These affections originating in or about the glands of Bartholin are similar to the anal fistula. They arise from abscesses (1) in the glands, (2) the cellular tissue surrounding them, (3) the pre-rectal vulva.

Dr. Edward M. Moore read a paper on *Recurring Luxations.* These he believed to be due to lacerations or weak spots in the capsular ligaments. Their occurrence in no way reflects on the surgeon.

Dr. Hyde read a paper on *The Difference in the Symptoms of Strangulated Oblique Inguinal Hernia*, giving a summary of the symptoms and indications for operation.

Dr. Gouley advocated early operations even if the diagnosis was obscure, believing that when no hernia was found the risk was slight.

Dr. Jamison read a paper on *Medicinal and Dietetic Thera-*

*peutics of Chronic Intestinal Catarrh.* Laxative remedies were recommended.

Dr. Garrish gave rectal injections of bismuth, but avoided oatmeal as a diet.

Dr. Purdy presented a paper entitled *A Cursory Review of the Epidemic and Endemic Diseases of Sullivan County, during the last Thirty-Four Years.* This was a very interesting sketch of the medical history of Sullivan County, New York, showing clearly the varying character of the different epidemics which had occurred, and the methods found most useful in controlling them.

Dr. Sabin described the *Removal of an Enterolith.* The stone was crushed and removed through the anus. Its weight was five ounces, and it was found to consist of petrified fæcal matter.

Dr. Van Zandt spoke of *Commercial Prescriptions.* It was noted that certain physicians had lent their names to advertise trade-preparations and this practice was severely condemned.

Dr. De Zanche read a paper upon *Prophylaxis,* urging upon physicians the need of inculcating hygienic principles, especially in the rural districts.

Dr. Baker declared the profession much too indifferent in these matters.

The following papers were read by title.

Dr. Alder. *Relation of Drink to Insanity.*

Dr. Tremaine. *Tumors of the Jaw.*

Dr. Bozeman. *Incontinence in Women from Hyperdilatation and Paralyzation of the Urethra.*

Dr. Leynde. *Skin-Flaps in Amputation and Umbilical Hæmorrhage in Infants.*

Dr. Fell presented a paper on *Some Etiological Factors in the Acne-form Diseases.* He attached much importance to the well-known fact of the presence of parasites called *Acarus* or *Domodex* in the pustules, and directed his treatment to the destruction of these with great success.

Dr. Janeway read the *Address on Pathology.* The study of micro-organisms seemed about to revolutionize pathological knowledge. Many obscure questions were now awaiting study and solution by those who were investigating the germs of diseases, particularly as to the possibilities of inoculation with attenuated virus, as a means of prevention. To the German pathologists he gave the greatest credit for original research. This was owing to the encouragement and assistance given by the government to scientific studies. Young men were induced to pursue scientific studies by the hope of attaining professorships. Here young men not only could not afford the time to become expert in these delicate manipulations, but the necessary apparatus cost twice as much as abroad. At the request of several present Dr. Janeway gave his views on the use of oxygen-gas in pneumonia. It was of great value in his experience in the cyanotic stages and should be administered whenever blueness of the skin showed itself.

Dr. Seymour read a paper on *A Case of Gall-Stones, Patent and Concealed. Exploratory Laparotomy—Autopsy eight weeks later.* Rather than entire removal of the gall-bladder, he recommended opening it and evacuating the contents, as a result of his observations in the case detailed.

Dr. North read a paper on the *Therapy of the Chlorides; Antisepsis, A Prominent and Important Factor in their Medical Action.* This was an exposition of the germ-theory of all infectious diseases. The efficacy of all medicines was due to their power in destroying micro-organisms. He claimed this particularly in the case of mercury, chloride of iron, etc.

Dr. Peters claimed great success for his method of treating diphtheria with bichloride of mercury gr. $\frac{1}{30}$ alternating with tincture of iron.

Dr. Newman read a paper on the *Causes of Failure in the Treatment of Urethral Stricture by Electrolysis.* Various causes of failure were alleged.

Dr. Meier praised the operation.

Dr. Manley agreed with the writer as to the difficulty of the operation.

Dr. Seymour read a paper on *The Diagnosis and Treatment of Pelvic Hæmatocele.* The diagnosis was stated to be obscure in the early stages but capable of being made, with certainty, later on.

When the hæmorrhage has been controlled, or has ceased spontaneously, absorption of the clot may occur, or it may soften and cause an abscess. Aspiration is recommended where a large collection exists.

The following papers were read by title:

Dr. Fitch. *Poisoning by two Grains of Strychnine.*

Dr. Rushmore. *Treatment of Abscess.*

Dr. Thayer. *Chronic Catarrhal Gastritis; Fatal from Sudden Enlargement of Thyroid Gland.*

Dr. Briggs. *Proper Disposition of Ashes, Swill, Etc.*

Dr. Leaning. *Ergot—Its Uses and Misuses.*

Dr. Stocton. *Nutrition in Lithæmia.*

Dr. Samuel W. Smith read a paper on *The History and Treatment of Thirty Cases of Diphtheria.* Of these cases five were fatal. The writer inclined to the theory that croup and diphtheria were one disease. His treatment consisted in the internal use of *Tr. Ferri. Mur.*, in glycerine, and potassa, chlorate, gargles of salt-water and spray of carbolized lime-water. Tracheotomy was not approved.

Dr. Hannan thought little was known of the real nature of this disease.

Dr. Harrison read a paper on *Peri-Uterine Hæmatoma.* The symptoms and cause of this form of trouble were carefully detailed.

Dr. Shrady read a report of a case of *Cancer of the Kidney.* Children have this disease more often than is known, the writer said. The diagnosis is plainer from the fact that enlargements of the spleen do not occur at that age. These growths form rapidly, displacing the other viscera and sometimes causing dyspnœa from pressure upon the diaphragm. Hæmaturia is often absent. The wandering of blue veins over the abdomen was noted as a symptom. Pain is usually present, sometimes constant but generally intermittent.

The following papers were read by title:

Dr. Manley. *Modern Aspect of Therapeutics.*

Dr. Hartmann. *The Vectisin Cross-Births, also Pessaries and their Uses.*

Dr. Gouley. *Contractures of the Bladder Consequent upon Cystitis.*

Dr. Sayre gave a demonstration of the method of applying a plaster-jacket.

Dr. Dennis spoke of *The Action of Micro-Organisms upon Surgical Wounds, with Demonstration.* Micro-organisms were classed as follows: (1) Sphero-bacteria; (2) Micro-bacteria; (3) Desmo-bacteria; (4) Spiro-bacteria; (5) Fungi.

